# The role of squamous cell carcinoma antigen (SCC Ag) in outcome prediction after concurrent chemoradiotherapy and treatment decisions for patients with cervical cancer

**DOI:** 10.1186/s13014-019-1355-4

**Published:** 2019-08-15

**Authors:** Jingxuan Fu, Weiping Wang, Yidan Wang, Chengeng Liu, Peichang Wang

**Affiliations:** 1 Department of Clinical Laboratory, Xuanwu Hospital, Capital Medical University, 45 ChangChun Road, Beijing, 100053 China; 2 Department of Radiation Oncology, Peking Union Medical College Hospital, Chinese Academy of Medical Sciences & Peking Union Medical College, Beijing, China

**Keywords:** Cervical cancer, Radiotherapy, Squamous cell carcinoma antigen

## Abstract

At present, the standard treatment approach for locally advanced cervical cancer is concurrent chemoradiotherapy (CCRT). An elevated pretreatment squamous cell carcinoma antigen (SCC Ag) level is associated with extensive tumors and poor survival for patients with cervical cancer treated with definitive CCRT. SCC Ag levels can be used to help physicians make decisions regarding surgery, avoiding the complications of double treatment modalities. Elevated SCC Ag is associated with radiotherapy resistance, and the rate of SCC Ag reduction during CCRT can predict tumor response after treatment. Moreover, the failure of SCC Ag levels to normalize posttreatment can predict tumor relapse, with a specificity higher than 70%, and adjuvant therapies should be considered for these patients. SCC Ag also plays an important role in the early detection of tumor relapse in patients with cervical cancer during follow-up after CCRT, with high sensitivity and good cost-effectiveness.

## Introduction

Serum squamous cell carcinoma antigen (SCC Ag) represents a subfraction of tumor-associated antigens related to squamous cell carcinoma and is widely used as a marker in squamous cell carcinoma of the head and neck, lung, and esophagus, among others. The most common histological type of cervical cancer is squamous cell carcinoma, accounting for more than 70% of cervical cancer cases in the United States [1] and for approximately 90% in China [2, 3]. At present, the standard treatment for locally advanced cervical cancer is definitive concurrent chemoradiotherapy (CCRT) [4], resulting in a 3-year disease-free survival (DFS) of 65–75% [2, 5]. SCC Ag is not documented in current guidelines or in routine clinical use for patients with cervical cancer. As outcomes after treatment are heterogeneous beyond available stratification factors such as the International Federation of Gynecology and Obstetrics (FIGO) stage and regional lymph node involvement [6], additional biomarkers, such as SCC Ag, need to be validated and incorporated into clinical practice. In the present study, we reviewed the literature on SCC Ag in predicting outcome or response after radiotherapy or CCRT in patients with locally advanced cervical cancer as well as the role of SCC Ag in treatment-related decisions for patients with early-FIGO-stage cervical cancer.

### Pretreatment SCC Ag and survival after CCRT

An elevated pretreatment SCC Ag level is associated with a more advanced stage [7–9], larger primary tumor size [7, 9, 10], regional nodal involvement [7, 8, 11, 12], and lymphovascular [8, 12] and deep stromal [8] infiltration in patients with cervical cancer.

It has been reported that high a pretreatment SCC Ag level is also associated with poor survival among patients treated with CCRT [7, 13–16]. Choi et al. reviewed 304 patients with cervical squamous cell carcinoma treated with CCRT and found that the recurrence-free survival rates of those with pretreatment SCC Ag levels < 4 ng/ml and ≥ 4 ng/ml were 80.2 and 56.6% (p < 0.001), respectively. Patients with elevated SCC Ag levels also suffered poor overall survival (OS) and high rates of local, regional and distant metastases [7]. Huang et al. found that an elevated pretreatment SCC Ag level was related to para-aortic lymph node relapse after CCRT [13]. Pretreatment SCC Ag levels have also been used for the risk stratification of patients with cervical cancer [14, 17]. Hong et al. reported an SCC Ag level > 2 ng/ml to be an independent risk factor for distant failure. The 5-year distant relapse-free survival rates for patients with SCC Ag levels < 2 ng/ml, stage IB-IIB disease, and negative lymph nodes and those with SCC Ag levels > 2 ng/ml, stage III, and positive lymph nodes were 83 and 43%, respectively [17]. In the study of Kang et al., the pretreatment SCC Ag level was an independent prognostic factor of distant recurrence and was incorporated into the nomogram predicting the probability of distant recurrence within 5 years [14].

### Pretreatment SCC Ag and treatment decisions

Pretreatment SCC Ag levels or risk stratification based on pretreatment SCC Ag levels might predict treatment failure or survival in patients with cervical cancer [7, 13–16], which can help physicians make clinical decisions. A high pretreatment SCC Ag level is associated with higher local-regional recurrence [7], distant metastasis [7, 14] and para-aortic recurrence [7, 13]. For patients with high SCC Ag levels, adjuvant therapies, such as neoadjuvant chemotherapy, consolidation chemotherapy [5, 18], high-dose brachytherapy, and extended-field irradiation [19], can be considered.

For patients with early-stage cervical cancer (FIGO stages IB1-IIA), radical hysterectomy (combined with adjuvant radiotherapy or CCRT when indicated) and definitive CCRT are equally effective treatment modalities. To avoid double-modality treatment, surgery should only be offered to patients with a low likelihood of undergoing adjuvant CCRT or radiotherapy. Pretreatment SCC Ag was associated with postoperative indications for adjuvant radiotherapy in patients with cervical cancer [8, 12, 20]. Reesink-Peters et al. reported that in those with early-stage cervical cancer and elevated pretreatment SCC Ag levels (> 1.9 ng/ml), 57% of patients at FIGO stage IB1 and 74% of patients at FIGO stage IB2/IIA had postoperative indications for adjuvant radiotherapy; in contrast, among those with normal pretreatment SCC Ag levels, 16% of patients at FIGO stage IB1 and 29% of patients at FIGO stage IB2/IIA had indications. Therefore, definitive CCRT rather than surgery are recommended for patients with FIGO stages IB-IIA and pretreatment SCC Ag levels > 1.9 ng/ml [20]. In the study by Xu et al., a pretreatment SCC Ag level ≥ 2.35 ng/ml was related to postoperative indications for radiotherapy, including lymph node metastasis, deep stromal infiltration, and primary tumor size ≥4 cm [8]. It should be noted that SCC Ag is just one of the judgment tools for assessing treatment policy, and it cannot be used alone. SCC Ag levels should be used in combination with other characteristics, such as tumor size, parametrial involvement, enlarged lymph nodes, and the general condition of the patient.

### SCC Ag during CCRT or radiotherapy

SCC Ag exists as two isoforms: SCC Ag 1 and SCC Ag 2. The expression of SCC Ag 1 is associated with radiation resistance [15, 21]. SCC Ag knockout reportedly increased the radiosensitivity of cervical tumor cell lines in vitro [15]. As a result, the level of SCC Ag or the rate of SCC Ag reduction during CCRT may predict tumor response or survival in patients with cervical cancer who are treated with CCRT [15, 22, 23]. Lee JH et al. found that the SCC Ag reduction rate during radiotherapy (before brachytherapy) was independently associated with OS (p = 0.003) in cervical cancer patients. The 5-year OS of patients with SCC Ag reduction rates ≤93.3% and > 93.3% were 74.9 and 95.4%, respectively (p < 0.0001). A scoring system was developed based on an SCC Ag reduction rate ≤ 93.3% (1 point), FIGO stage > II (1 point), and a tumor volume reduction rate ≤ 87% (1 point). The 5-year OS for patients with scores of 0, 1, 2, and 3 were 98.6, 95.3, 74.2, and 45.0%, respectively (p < 0.0001, [22]). Furthermore, Markovina et al. found persistently elevated SCC Ag levels during CCRT to be an independent risk factor for tumor recurrence (p = 0.0046) and death (P = 0.015, [15]). In the study by Lee KC et al., a significant correlation between the rate of primary tumor volume reduction and that of SCC Ag reduction during definitive CCRT (correlation coefficient 0.550, *p* < 0.001) was found for patients with cervical cancer. The primary tumor volume reduction rate was associated with progression-free survival [23].

### Posttreatment SCC Ag

In contrast to surgical outcomes, the regression of tumors after CCRT may require more than 3 months [24], and it is difficult to identify whether a patient has achieved or will achieve a complete response via gynecologic examination, MRI [24] or even biopsy. Thus, assessing SCC Ag levels has the potential to help physicians make decisions [16, 25–27]. Kawaguchi et al. reviewed 116 patients with cervical cancer treated with definitive radiotherapy or CCRT. The optimal cutoff point for the posttreatment SCC Ag level (one month after the completion of treatment) was 1.15 ng/ml (sensitivity 80.0%, specificity 74.0%). For patients with posttreatment SCC Ag levels < 1.15 ng/ml and ≥ 1.15 ng/ml, the 3-year OS rates were 90.7 and 36.6% (p < 0.001), and the 3-year progression-free survival (PFS) were 74.7 and 19.5% (p < 0.001), respectively [25]. Ryu et al. reviewed 783 patients with cervical squamous cell carcinoma and found that the optimal cutoff point for the posttreatment SCC Ag level in predicting recurrence was 0.9 ng/ml (sensitivity 44.2%, specificity 72.0%). The posttreatment SCC Ag level was also an independent prognostic factor for DFS (p = 0.003, [16]). In the study by Olsen et al., failure of posttreatment SCC Ag levels to normalize (< 2.2 ng/ml, at the completion of treatment) in patients with cervical cancer treated with CCRT was associated with an incomplete metabolic response on 3-month posttreatment positron emission tomography/computed tomography imaging and a decreased PFS. The 2-year PFS rates were 62 and 0% for patients with normalized posttreatment SCC Ag and elevated posttreatment SCC Ag levels, respectively (p = 0.0004, [27]). Additionally, a meta-analysis revealed that posttreatment SCC Ag levels can predict recurrence and survival in patients with cervical cancer treated with radiotherapy, CCRT or surgery [26].

**Fig. 1:**
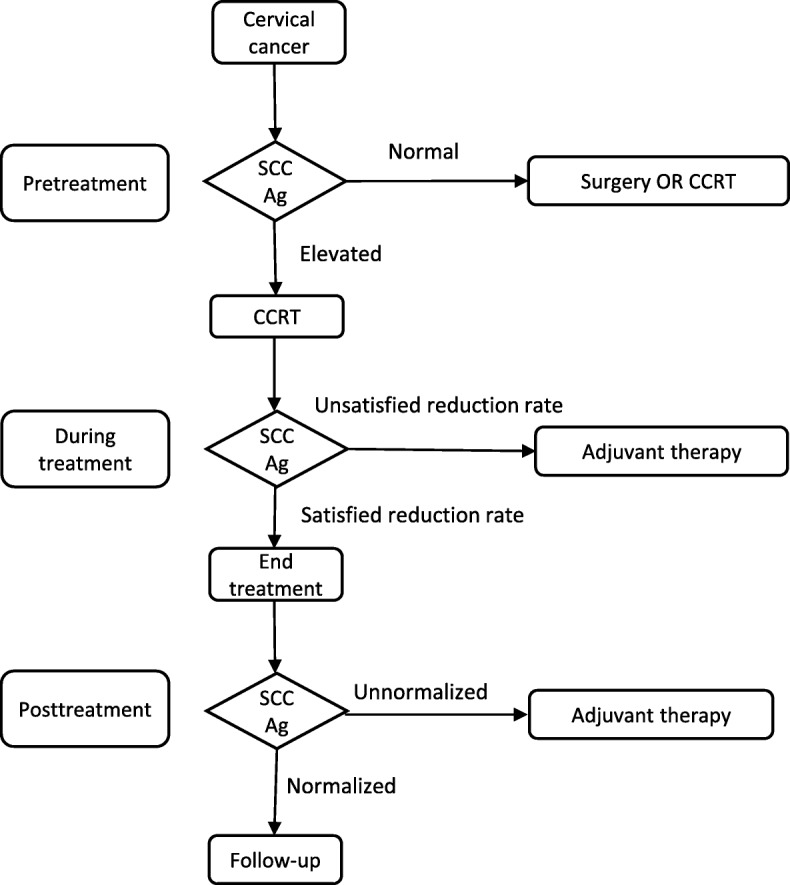
The roles of SCC Ag in decision-making for patients with cervical cancer. Abbreviations: *CCRT* concurrent chemoradiotherapy, *SCC Ag* squamous cell carcinoma antigen

Although the cutoff points varied, including 0.9 ng/ml, 1.15 ng/ml and 2.2 ng/ml, the survival rates of patients with elevated posttreatment SCC Ag levels were poor in the above studies [16, 25–27]. The PFS rates of patients with elevated posttreatment SCC Ag levels were less than 20%, and the specificities were higher than 70% [16, 25]. These results indicate that adjuvant chemotherapy [5, 18], additional brachytherapy, salvage hysterectomy or other currently available adjuvant therapies are reasonable. Despite no definitive evidence that these adjuvant therapies are effective in improving outcomes for cervical cancer patients, they are needed for patients known to have a high risk of tumor recurrence. An ongoing trial, the OUTBACK trial (NCT01414608), is a randomized phase III trial comparing CCRT followed by adjuvant chemotherapy (carboplatin and paclitaxel) and CCRT alone for patients with locally advanced cervical cancer. If the OUTBACK trial demonstrates that adjuvant chemotherapy improves survival, we will have definitive evidence to recommend adjuvant chemotherapy for patients with elevated posttreatment SCC Ag levels. If adjuvant therapies are not utilized, extensive follow-up should be considered. Figure 1 summarized the roles of SCC Ag levels in decision-making before, during and after treatment in patients with cervical cancer.

### SCC Ag for the surveillance of cervical cancer treated with CCRT

For patients with cervical cancer treated with definitive CCRT or radiotherapy, the level of SCC Ag normalized in most patients who had a complete response [27]. However, 20–30% of patients suffered from tumor relapse during follow-up [2, 7, 16, 22, 28]. Early detection of tumor relapse has a significant impact on prognosis and may improve the survival of patients [29]. Among cervical cancer patients with tumor relapse, 62.3–82.4% had elevated SCC Ag levels before the diagnosis of tumor relapse [28, 30, 31].

In the surveillance of cervical cancer, the Society of Gynecologic Oncology (SGO) [32] and National Comprehensive Cancer Network guidelines [4] recommend a medical history, a physical examination, cervical/vaginal cytology and imaging as indicated based on symptoms or suspicion for recurrence. However, assessment of SCC Ag is not currently recommended. Oh et al. analyzed 53 patients with locally advanced cervical cancer who were primarily treated with definitive CCRT or radiotherapy and experienced tumor relapse and found that adding SCC Ag assessment to the basic follow-up protocol recommended by the SGO may improve sensitivity for detecting tumor relapse. The sensitivity of the basic protocol and the basic protocol plus the SCC Ag protocol were 49.1 and 88.7%, respectively (p < 0.001). Early diagnosis of tumor relapse that can be treated by salvage therapy, which may lead to better survival [30]. Another study by Oh et al. demonstrated that the optimal cutoff value of the SCC Ag level for detecting tumor relapse was 2 ng/ml [33]. Yoon et al. reviewed the records of 116 patients with cervical cancer who were treated with CCRT and found that 18 developed recurrent disease. The change in SCC Ag (ΔSCC Ag), which is defined as the difference between the last elevated value and the value immediately before elevation, might accurately predict tumor relapse. The optimal cutoff value of ΔSCC Ag was 0.95 ng/ml. The true positive and false positive rates were 75 and 11%, respectively [34]. In a study by Forni et al., the sensitivity, positive predictive value and negative predictive value of SCC Ag were 79.1, 89.5 and 90.7%, respectively, during the follow-up of patients with cervical cancer treated with radiotherapy or CCRT; the cutoff value was 1.4 ng/ml. In a study by Hu et al., the sensitivity and positive predictive value of SCC Ag in detecting tumor recurrence of cervical cancer were 72.1 and 96.9%, respectively [35]. Compared with the complete follow-up protocol, the recurrence miss rate of the simplified approach (SCC Ag plus gynecologic examination) was 2.2%. The cost-effectiveness profile of the simplified approach was better than that of the standard approach [31].

Overall, SCC Ag assessment is a useful tool that can be used in the follow-up of patients with cervical cancer treated with CCRT or radiotherapy [9, 30, 33–35].

## Conclusions

SCC Ag plays an important role in patients with cervical cancer treated with definitive CCRT or radiotherapy. An elevated pretreatment SCC Ag level is associated with extensive tumors and poor survival in patients with cervical cancer treated with definitive CCRT or radiotherapy. SCC Ag levels can be used to help physicians make decisions regarding surgery. Elevated SCC Ag levels are associated with radiation resistance, and the SCC Ag reduction rate during CCRT can predict the tumor response after treatment. Failure of posttreatment SCC Ag levels to normalize might also predict tumor relapse, with a specificity higher than 70%. For these patients, adjuvant therapies should be considered. SCC Ag assessment has high sensitivity and good cost-effectiveness for the early detection of tumor relapse during follow-up.

## Data Availability

Data sharing is not applicable to this article because no datasets were generated during the study.

## Abbreviations

CCRT: Concurrent chemoradiotherapy;
DFS: Disease-free survival
FIGO: International Federation of Gynecology and Obstetrics
NACT: neoadjuvant chemotherapy
OS: Overall survival
PFS: progression-free survival
SCC Ag: Squamous cell carcinoma antigen
SGO: Society of Gynecologic Oncology
